# *SCGB1A1* rs3741240 variant downregulates CC16: a molecular insight into COPD pathogenesis in Indian population

**DOI:** 10.3389/fimmu.2025.1689340

**Published:** 2025-12-12

**Authors:** Nasima Sultana, Himani Adhikari, Achintya Mohan Goswami, Amalesh Mondal, Indranil Ganai, Himani Biswas, Asif Iqbal, Aratrika Das, Saibal Moitra, Sanjoy Podder

**Affiliations:** 1 Ecology and Allergology Laboratory, Department of Zoology, The University of Burdwan, Burdwan, India; 2 Department of Physiology, Krishnagar Government College, Krishnagar, India; 3 Department of Physiology, Katwa College, Katwa, India; 4 Post Graduate Department of Zoology, Lady Brabourne College, Kolkata, India; 5 Apollo Multispecialty Hospitals, Kolkata, India

**Keywords:** COPD, *SCGB1A1*, SNPs, bronchial epithelial cell, immunofluorescence, gene expression, immune regulation, precision immunology

## Abstract

**Background:**

Chronic Obstructive Pulmonary Disease (COPD) is a progressive inflammatory lung disorder influenced by environmental and genetic factors. The rs3741240 polymorphism in the *SCGB1A1* gene, which encodes the anti-inflammatory protein CC16, is considered a genetic marker of COPD susceptibility.

**Objective:**

This study aimed to investigate the functional significance of the *SCGB1A1* rs3741240 polymorphism in 224 COPD patients and 194 controls in the West Bengal population, India.

**Methods:**

Genotyping of rs3741240 was performed using Polymerase Chain Reaction-Restriction Fragment Length Polymorphism (PCR-RFLP). SCGB1A1 mRNA levels were quantified in healthy controls and among different genotypes using Real-Time PCR. Protein expression was assessed using Western blotting. *In silico* analyses identified miRNAs and transcription factors that target the *SCGB1A1* promoter using the miRDB, RNA22 v2, and hTFtarget web servers. Structural modeling and docking studies were conducted to analyze miRNA-mRNA-AGO2 and TF-promoter interactions using the I-TASSER, SWISS-MODEL, GalaxyGemini, RNAfold, RNA COMPOSER, HDOCK, and DNAproDB web servers.

**Results:**

The genotype and allele frequencies of the 38AA risk genotype were more prevalent in COPD patients than in controls (P = 0.01 and 0.03, respectively). Patients with 38AA genotype exhibited significantly lower FEV1 and FEV1/FVC (P <0.0001). *SCGB1A1* mRNA and protein expression were significantly reduced in patients bearing the 38AA genotype compared to those bearing the GA and GG genotypes. Bioinformatics analyses suggested that reduced CC16 expression for rs3741240 might be mediated by miRNA hsa-miR-11181-3p and the transcription factors SMAD1, HAND1, and HAND2.

**Conclusion:**

This study is the first to reports in an Indian population that *SCGB1A1* rs3741240 is linked to COPD via CC16 downregulation, revealing a key pathogenic mechanism and potential for precision medicine in management.

## Introduction

Chronic obstructive pulmonary disease (COPD) is a group of progressive inflammatory lung conditions, primarily chronic bronchitis and emphysema (1). It leads to airflow obstruction and breathing difficulties and is associated with an increased risk of cardiovascular and other pulmonary complications (2). Globally, an estimated 3.23 million people die from COPD each year, making it the third leading cause of death (3). As COPD is both progressive and currently incurable, early diagnosis and the development of effective treatments are essential (4). While long-term exposure to cigarette smoke is the most common cause of COPD, genetic factors play a crucial role in individual susceptibility to COPD (5). One such candidate is the single nucleotide polymorphism (SNP) rs3741240 in the *SCGB1A1* gene, which encodes Clara Cell Secretory Protein (CC16), a key anti-inflammatory molecule in the airway epithelium (6). CC16, a member of the secretoglobin family, is the most abundant protein found in normal airway secretions and is primarily produced by non-ciliated club cells of the distal airway epithelium (7). Other non-ciliated epithelial cells also contribute to its production at lower levels (8).

Lin and colleagues demonstrated that CC16 promotes epithelial repair and mitigates both inflammation and pyroptosis in PM_2.5_-exposed asthmatic mice (9). Furthermore, a 2022 cohort study of U.S. veterans identified interactions between CC16 levels, smoking status, and lung function, showing that elevated CC16 levels in former smokers were associated with improvements in FEV_1_/FVC ratios (8). Thus, research over several decades suggests that COPD, cigarette smoke, and CC16 are strongly associated with and collectively affect lung function. A longitudinal birth cohort study demonstrated that reduced CC16 levels during childhood are associated with impaired lung function and increased airway hyperreactivity that persists into adulthood (10). Chen et al. (11) reported reduced serum and BALF CC16 levels in COPD patients and cigarette smoke-exposed monkeys. Laucho-Contreras et al. (12) found that decreased CC16 correlates with greater airflow obstruction.

The SNP rs3741240 in the 5′ untranslated region/promoter of the *SCGB1A1* gene has been identified as a key regulatory variant significantly associated with CC16 expression levels (13). Furthermore, the *SCGB1A1* rs3741240 polymorphism has been identified as a major genetic determinant influencing circulating CC16 levels, lung function decline, and susceptibility to COPD (14–16).

Although growing global evidence links *SCGB1A1* variants and CC16 levels to COPD pathogenesis, a significant geographical and ethnic gap persists in this area of research. Most studies investigating the *SCGB1A1* rs3741240 variant and its impact on CC16 expression have been conducted in Western populations, with minimal data available from Indian cohorts, despite India’s distinct genetic background, diverse environmental exposures, and high COPD burden. 

Therefore, this study aimed to evaluate the relationship between the *SCGB1A1* rs3741240 polymorphism and CC16 downregulation and to examine its potential role in the molecular pathogenesis of COPD in the West Bengal population.

## Materials and methods

### Study subject

This study was approved by the Clinical Research Ethics Committee of the Allergy and Asthma Research Centre, West Bengal, India (CREC-AARC Ref: 62/204). Informed consent was obtained from all the participants. Epidemiological information, including age, sex, weight, height, residence, occupation, and smoking habits, was collected using a standard questionnaire. A total of 447 individuals, both male and female, aged 20 years or older were selected for this study. Among them, 28 participants were excluded due to other respiratory diseases, pregnancy, or failure to provide a written informed consent. Finally, of the 418 selected participants, 224 were clinically diagnosed with COPD.

### Subject classification

The patient groups were further classified into young (20–50 years) and old (>50 years) COPD groups according to the Global Initiative for Chronic Obstructive Lung Disease (GOLD) criteria, 2025 (17). Both patient and control participants were classified according to sex (male and female), age (20–50 and >50 years), smoking status (current smoker, occasional smoker, ex-smoker, and non-smoker), and disease severity (mild, moderate, severe, and very severe).

### Pulmonary function test

Hospital pulmonologists diagnosed COPD based on clinical examination and spirometry (GOLD criteria, 2025) (17). COPD was confirmed by a post-bronchodilator FEV_1_/FVC <0.7. The severity of COPD is classified based on post-bronchodilator FEV_1_ as follows: ≥80% (mild, GOLD 1), 50%–79% (moderate, GOLD 2), 30%–49% (severe, GOLD 3), and <30% (very severe, GOLD 4) (GOLD, 2025) (17).

### Blood collection

Peripheral blood (2 mL) was collected from both the patients and the control group in EDTA non-vacuum blood collection vials, and serum was separated by centrifugation (1,200 rpm, 10 min). Serum-free samples were stored at 4 °C for genotyping.

### DNA extraction and genotyping

**Figure 1:**
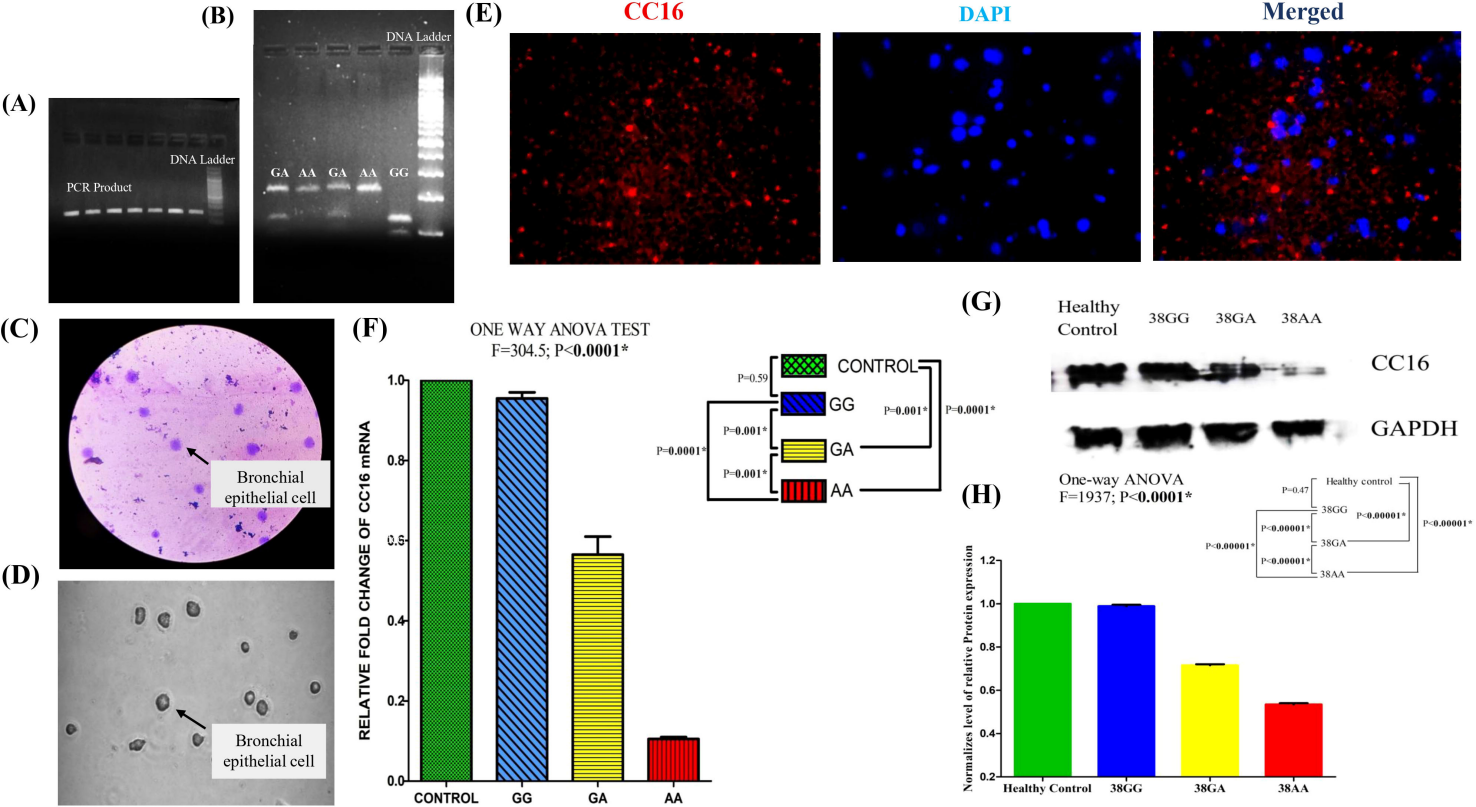
Comprehensive analysis of cellular morphology, *SCGB1A1* gene expression, and protein localization: **(A)** PCR amplification of *SCGB1A1* gene polymorphism (224 bp). **(B)** RFLP analysis of rs3741240 PCR product. **(C)** Giemsa staining reveals characteristic epithelial morphology, and well-defined nuclei. **(D)** Brightfield microscopy image showing the typical morphology of bronchial epithelial cells. **(E)** Immunofluorescence staining for CC16 (red) and DAPI (blue). CC16 is localized in the cytoplasm, consistent with secretory epithelial cell phenotype, while DAPI stains the nuclei. **(F)** mRNA expression level of *SCGB1A1* using 2^−ΔΔCT^ method in healthy control and patients bearing different genotypes 38GG, 38GA, and 38AA. **(G)** Western blot analysis for CC16 protein of COPD patients with three different polymorphic variants and healthy controls. Human GAPDH protein was used as loading control. **(H)** Differential protein expression of *SCGB1A1* in control and different polymorphic genotype of patient by One-way ANOVA followed by *Post hoc* Tukey analysis with Bonferroni correction. (^*^P value significant).

Genomic DNA was extracted from blood samples using the QIAamp DNA Blood Mini Kit (Qiagen, Germany) according to the manufacturer’s instructions. SNPs in the *SCGB1A1* gene were identified by polymerase chain reaction (PCR) followed by restriction fragment length polymorphism (RFLP) analysis (**Figures 1A, B**). The PCR primers, amplicon size, restriction enzymes, and expected RFLP fragment patterns are detailed in **Table 1**.

**Table 1:** PCR conditions and restriction fragments of *SCGB1A1* rs3741240 polymorphism.

SNP	Functional position	Primer sequence and PCR product size	PCR condition	Restriction enzyme, site, fragments and genotypes
SCGB1A1 rs3741240	Promoter +38 G>A	Forward: 5’- GCAGTATCTTATGTAGAGCCCT -3’ Reverse: 5’- TCTCTGAGCACTCACCGGAGCT -3’ Product size- 224 bp	95 °C for 5 min, followed by 35 cycles at 95 °C for 30 s, 61°C for 30 s, 72 °C for 1 min, and a final extension at 72 °C for 10 min	Restriction enzyme- Sau 96 I Restriction site- G_GNCC (N = A, T, G, C) GG- 129, 95 bp AA- 224 bp GA- 224, 129 and 95 bp

### DNA sequencing

To validate the RFLP results, representative PCR products from each of the three genotypes for the selected polymorphisms were subjected to Sanger sequencing (ABI 3500 Genetic Analyzer) according to the manufacturer’s standard protocol.

### Bronchoscopic sampling of bronchial epithelial cells

Bronchial epithelial cells (BECs) were collected from selected participants via bronchoscopy using a sterile cytology brush. Cells were released from the pellet by vigorous agitation, filtered through a 70-µm strainer to remove mucus and debris, and centrifuged at 14,000 rpm for 10 min at 4 °C. The pellet was resuspended in PBS for RNA and protein extraction.

### Giemsa and immunofluorescence staining of BECs

BECs were fixed and stained with Giemsa (Sigma-Aldrich; 1:20) to assess their morphology. The stained cells were mounted with DPX and examined under a light microscope at ×40 magnification. For immunofluorescence staining, BEC pellets were washed with PBS, fixed in 2% paraformaldehyde (20 min, room temperature), and permeabilized with 0.2% Triton X-100 in PBS for 10 min. Cells were incubated overnight at 4 °C with CC16 rabbit polyclonal antibody (ABclonal; A16997; 1:100), washed thrice with PBS, and incubated with PE Donkey anti-rabbit IgG (Biolegend; 406421, 1:200) for 1 h at room temperature in the dark. Nuclei were stained with DAPI (1 µg/mL, 5 min), coverslips were mounted with ProLong™ Gold Antifade (Invitrogen), and images were captured using confocal microscopy. ImageJ was used to analyze CC16 localization.

### RNA and protein extraction

Bronchial epithelial cells (BECs) from COPD patients and controls were processed for RNA and protein isolation. BECs suspended in 1× PBS were used for total RNA extraction using TRIzol reagent (Invitrogen, USA) according to the manufacturer’s instructions. RNA yield and purity were assessed using a NanoDrop spectrophotometer (Thermo Fisher), and RNA integrity was verified by agarose gel electrophoresis. DNase I (Qiagen) treatment was performed to remove genomic DNA before reverse transcription.

For protein isolation, BECs were lysed in RIPA buffer with protease inhibitor (50×, Promega), incubated on ice (20 min), centrifuged (12,000 rpm, for 10 min at 4 °C), and the supernatants were collected. Protein concentration was measured using the Bradford assay (Bio-Rad).

### Quantitative RT-PCR analysis

Real-time reverse transcription polymerase chain reaction (RT-PCR) was performed using 2 μL of RNA with the Hi-Quanti One Step Probe-Based RT-PCR Kit (HiMedia, India), according to the manufacturer’s protocol. *SCGB1A1* was amplified using the following primers: forward 5′ ATGAAGATCGCCATCACAATCA 3′ and reverse 5′ GAATCTTAAATCTTGCTTACACAG 3′. The RT-PCR conditions were as follows: 95 °C for 1 min, followed by 40 cycles of 95 °C for 10 s and 60 °C for 40 s. Expression was quantified using the 2^−ΔΔCT^ method (18), with GAPDH as the loading control. Each reaction was conducted in triplicate across three biological replicates for the 38GG, 38GA, and 38AA genotypes of rs3741240 in a case–control design.

### Western blot analysis

Western blotting was performed on CC16 protein extracts from BECs of COPD patients and controls, using three representative samples per *SCGB1A1* variant. Proteins (20 µg –40 µg) were separated on 10% SDS-PAGE, transferred to nitrocellulose membranes, and blocked with 5% BSA (1 h, room temperature). The membranes were incubated overnight at 4 °C with primary antibodies: SCGB1A1 Rabbit pAb (ABclonal; A16997, 1:1,000) and anti-GAPDH (Bio Bharati Life Science; BB-AB0060, 1:2,000), followed by HRP-conjugated secondary antibody (1:5,000, 1 h, room temperature). Bands were visualized and quantified using ImageJ software, with CC16 normalized to GAPDH.

### *In silico* study

#### Identification of microRNAs targeting *SCGB1A1* promoter

We used the miRDB web server to identify microRNAs (miRNAs) that target the *SCGB1A1* promoter (19). The query promoter sequence (both wild type and mutant) of *SCGB1A1* was provided as input, followed by the selection of the 5’ UTR from the dropdown list. miRDB predicted the miRNAs targeting the 5’ UTR of both the wild-type and mutant *SCGB1A1* promoters. The RNA22 v2 web server was used to obtain miRNA–target interaction (MTI) data for the target miRNAs in both wild-type and mutant *SCGB1A1* promoters (20, 21). The FASTA sequences of miRNAs and both the wild-type and mutant *SCGB1A1* promoters were used as inputs in RNA22 v2 to obtain MTI details.

#### 2D and 3D structural prediction of miRNA–mRNA duplexes

We used the RNAfold web server (http://rna.tbi.univie.ac.at/cgi-bin/RNAfold.cgi) to obtain the optimal secondary structure of both wild-type and mutant miRNA–mRNA duplexes. Dot bracket notation of the miRNA–mRNA duplexes was also obtained from the server. These dot-bracket notations were used as inputs for the RNA COMPOSER web server to obtain the 3D structures of both wild-type and mutant miRNA–mRNA duplexes (22).

#### Retrieval of human AGO2 structure and its preparation

The 3D crystal structure of the human AGO2 (hAGO2) protein (PDB ID: 4F3T) was retrieved from the Protein Data Bank. The hAGO2 protein was prepared by removing the heteroatoms and water molecules using BIOVIA Discovery Studio 2020. To address the missing atoms observed in hAGO2, we homology-modeled the protein in I-TASSER using cleaned hAGO2 as a template. The newly modeled hAGO2 was energy-minimized for docking using Swiss-PDBViewer 4.1.0.

#### Docking study between miRNA–mRNA duplexes and human AGO2 protein

The HDOCK web server was used for docking between the miRNA–mRNA duplexes and hAGO2 protein (23). We chose the miRNA–mRNA duplex–HAGO2 complex based on the best docking scores provided by the server algorithm.

#### Identification of transcription factors targeting the *SCGB1A1* promoter

We used the hTFtarget web server to identify TFs targeting the selected nucleotide stretch (ACCAGAGACGGGCCAGAGCAT) in both the wild-type and mutant *SCGB1A1* promoters (24).

#### Modeling of the DNA bonding domain of predicted TFs and the selected nucleotide stretch of the *SCGB1A1* promoter

The DNA-binding domains of the predicted TFs were modeled using template-guided homology modeling in SWISS-MODEL. The modeled DNA bonding domain of the predicted TFs was dimerized using the GalaxyGemini web server, while both wild-type and mutant *SCGB1A1* promoters (selected nucleotide stretch) were constructed using Discovery Studio (25).

#### Docking study between predicted TFs and *SCGB1A1* promoter with selected nucleotide stretch

We used the HDOCK web server to dock the predicted TFs with both the wild-type and mutant *SCGB1A1* promoters (23).

#### DNA–protein interaction analyses

The DNAproDB web server was used for interaction analyses of DNA (*SCGB1A1* promoter)-protein (TF) docked complexes (26).

### Data analysis

Categorical data are presented as numbers with percentages, and continuous data are expressed as median ± standard deviation (SD). The Pearson’s chi-square test was used to analyze categorical variables. For continuous variables, the independent *t*-test was applied to normally distributed data, and the Mann–Whitney *U* test was used for non-normally distributed data. For variables with non-normal distributions, the interquartile range (IQR) represents the middle 50% of the data and is calculated as the difference between the third (Q3) and first (Q1) quartiles. Data points falling outside the range (Q1 − 1.5 × IQR, Q3 + 1.5 × IQR) were considered as potential outliers. The contingency chi-square test was used to assess the genotype and allele distributions across the study groups. Risk analysis for additive, recessive, and dominant genetic models was performed using odds ratios (OR). The Hardy–Weinberg equilibrium (HWE) was evaluated using the one-degree-of-freedom chi-square goodness-of-fit test. A Generalized Linear Model (GLM) was employed with binomial logit and quasi-Poisson log links. In these models, risk genotypes were treated as dependent variables, whereas disease severity, age, and residence were included as independent predictors. A one-way ANOVA followed by Tukey’s Honestly Significant Difference (HSD) test was used to examine the association between FEV_1_, FEV_1_/FVC ratio, and genetic polymorphisms. Fold changes in mRNA and protein expression levels of *SCGB1A1* among controls and patient genotypes were analyzed using one-way ANOVA followed by *post hoc* Tukey analysis with Bonferroni correction. All statistical analyses were performed using GraphPad Prism version 7 (San Diego, CA, USA), with a significance threshold set at *P <*0.05.

## Results

### Study subjects

The demographic and clinical characteristics of the 224 patients and 194 controls are presented in **Table 2**. Significant differences were observed between the patients and controls in terms of age, residence, smoking habits, and disease severity. Among the 224 patients, 33.93% were classified as having Young COPD, while 66.07% had Old COPD. In this study, the majority of patients were from rural areas (41.96%), followed by urban and semi-urban regions. In terms of disease severity, 27.23% had mild COPD, 29.91% had moderate COPD, 23.21% had severe COPD, and 19.64% had very severe COPD. Compared to controls, patients showed significant differences in key pulmonary function parameters, including fractional exhaled nitric oxide (FeNO), diffusing capacity of the lungs for carbon monoxide (DLco), and forced expiratory volume in 1 s (FEV1).

**Table 2:** Demographic and clinical characteristics of case and control subjects.

Sl. No	Parameters	Category	Case (n = 224)	Control (n = 194)	P value
1.	Age, n (%)	20–50	76 (33.93%)	119 (61.34%)	0.0001^*^
		>50	148 (66.07%)	75 (38.66%)	
2.	Sex, n (%)	Males	118 (60.82%)	102 (52.58%)	0.10
		Females	106 (54.64%)	92 (47.42%)	
3.	Family history	Paternal	48 (21.43%)	Nil	–
		Maternal	72 (32.14%)	Nil	
		P + M	25 (11.16%)	Nil	
		Absent	79 (35.27%)	194 (100%)	
4.	Weight, mean ± SD (kg)	–	63.16 ± 10.24 (61.81–64.51)	63.89 ± 10.24 (62.44–65.34)	0.45
5.	Height, mean ± SD (cm)	–	166.97 ± 8.95 (165.79–168.15)	167.06 ± 8.89 (165.80–168.32)	0.92
6.	Residence, n (%)	Urban	44 (19.64%)	88 (45.36%)	0.0003^*^
		Semi-urban	86 (38.39%)	59 (30.41%)	
		Rural	94 (41.96%)	47 (24.23%)	
7.	Occupation, n (%)	Industry	54 (24.11%)	46 (23.71%)	0.98
		Transport	47 (20.98%)	38 (19.59%)	
		Agriculture	31 (13.84%)	26 (13.40%)	
		Service	57 (25.45%)	47 (24.23%)	
		Unemployed	35 (15.63%)	37 (19.07%)	
8.	Smoking Habit, n (%)	Current Smoker	87 (38.84%)	17 (8.76%)	<0.0001^*^
		Occasional Smoker	28 (12.50%)	23 (11.86%)	
		Ex-smoker	16 (7.14%)	21 (10.82%)	
		Non-smoker	93 (41.52%)	133 (68.56%)	
10.	Disease severity, n (%)	Mild (GOLD 1)	61 (27.23%)	–	–
		Moderate (GOLD 2)	67 (29.91%)		
		Severe (GOLD 3)	52 (23.21%)		
		Very severe (GOLD 4)	44 (19.64%)		
11.	DLco, mean ± SD (%)	–	58.56 ± 25.83 (55.16–61.96)	97.94 ± 11.23 (96.35–99.53)	<.00001^*^
12.	FeNO, median (IQR) ppb	–	36	13	<.00001^*^
13.	FVC, mean ± SD (Liters)	–	3.69 ± 0.25 (3.66–3.72)	3.66 ± 0.25 (3.63–3.69)	0.06
14.	FEV1, mean ± SD (Liters)	–	2.04 ± 0.64 (1.96–2.12)	2.90 ± 0.46 (2.82–2.98)	<.00001^*^

(*P value significant).

### Genotyping by PCR-RFLP

Genotyping of the SCGB1A1 rs3741240 variant was confirmed using PCR-RFLP analysis. The PCR product (224 bp) was digested to yield distinct fragments: GG = 129 bp + 95 bp, GA = 224 bp + 129 bp + 95 bp, and AA = 224 bp. Representative PCR and RFLP gel images are shown in **Figures 1A, B**.

### Allele and genotype analysis

The allele and genotype distributions of the rs3741240 polymorphism in our study population are shown in **Table 3**. The SNP did not show a significant deviation from HWE in the control population (χ^2^ = 0.54, df = 1). In our study, we found significant differences in genotype frequency under the additive (P = 0.01), recessive (P = 0.01), and dominant (P = 0.03) models between the case and control populations. The frequency of the AA risk genotype was significantly higher in the COPD case population than in the control population (OR = 6.43; P = 0.01). According to sex, age, and residence, and disease severity, the distribution of genotypes in both COPD cases and control subjects demonstrated significant differences between males and females (χ^2^ = 24.79; P = 0.0004), Young and Old COPD cases (χ^2^ = 24.37; P = 0.0004), rural, urban, and semi-urban areas (χ^2^ = 30.42; P = 0.001), as well as mild, moderate, severe, and very severe patients (χ^2^ = 28.00; P = 0.001), respectively. In female patients, the AA risk genotype was significantly associated with a higher COPD risk than in male patients (OR = 25.10; P = 0.002). The risk genotype in old COPD cases was significantly associated with a higher COPD risk than in young COPD cases (OR = 45.61; P = 0.01). Additionally, risk genotypes were associated with a higher risk of COPD in semi-urban and rural areas than in urban areas (OR = 7.73; P = 0.01 and OR = 2.06; P = 0.02, respectively). Furthermore, the risk genotype was associated with a higher risk of COPD in patients with severe and very severe disease than in those with mild or moderate disease (OR = 10.85; P = 0.0002 and OR = 10.08; P = 0.0004, respectively). The Power of the sample size was also calculated to identify statistically significant risk factors [Power (1 − β) = 88.63%].

**Table 3:** Genotypic frequency distribution of SCGB1A1 rs3741240 polymorphism in patients and control.

Genotype	Model	Case (n = 224)				Control (n = 194)			Chi Square Value (P value)	OR (95% CI)				P value				MAF
I. Overall distribution																		
GG	Additive	102 (45.74%)				121 (62.37%)			10.29 (P = 0.01*)	1.00				–				0.21
GA		91 (40.81%)				67 (34.54%)				1.61 [0.89–2.92]				0.11				
AA		31 (13.90%)				6 (3.09%)				6.43 [1.74–23.70]				0.01*				
GG+GA	Recessive	193 (86.16%)				188 (96.91%)			6.43 (P = 0.01*)	1.00				–				
AA		31 (13.90%)				6 (3.09%)				5.26 [1.46–18.94]				0.01*				
GG	Dominant	102 (45.74%)				121 (62.37%)			4.53 (P = 0.03*)	1.00				–				
GA+AA		122 (54.46%)				73 (37.63%)				1.92 [1.09–3.37]				0.01*				
G		295 (65.85%)				309 (79.64%)			4.97 (P = 0.03*)	1.00				–				
A		153 (34.15%)				79 (20.36%)				2.06 [1.09–3.91]				0.03*				

II. Distribution according to sex																	
Genotype		Male (n = 118)		Female (n = 106)		Male (n = 102)		Female (n = 92)		Male		Female		Male		Female	
GG	–	57 (48.31%)		45 (42.45%)		64 (62.75%)		57 (61.95%)	24.79 (P = 0.0004^*^)	1.00		1.00		–		–	
GA		48 (40.68%)		43 (40.57%)		33 (32.35%)		34 (36.96%)		1.68 [0.93 - 3.05]		1.64 [0.90-2.96]		0.09		0.10	
AA		13 (11.01%)		18 (16.98%)		5 (4.90%)		1 (1.10%)		2.89 [0.94 - 8.87]		25.10 [3.22-195.82]		0.06		0.002^*^	

III. Distribution according to age (Years)																	
Genotype		Young COPD case [20–50] (n = 76)		Old COPD case [>50] (n = 148)		20-50 (n = 119)		>50 (n = 75)		20–50		>50		20-50		>50	
GG	–	37 (48.68%)		65 (43.91%)		75 (63.03%)		46 (61.33%)	24.37 (P = 0.0004^*^)	1.00		1.00		–		–	
GA		31 (40.79%)		60 (40.54%)		38 (31.93%)		29 (38.67%)		1.65 [0.91–2.98]		1.46 [0.81–2.62]		0.10		0.21	
AA		8 (10.53%)		23 (15.54%)		6 (5.04%)		0 (0.00%)		2.82 [0.92–8.68]		45.61 [2.67–780.49]		0.07		0.01^*^	

IV. Distribution according to Residence																	
Genotype		Urban (n = 44)	Semi-urban (n = 86)		Rural (n = 94)	Urban (n = 88)	Semi-urban (n = 59)	Rural (n = 47)		Urban		Semi-urban	Rural	Urban		Semi-urban	Rural
GG	–	22 (50.00%)	39 (45.35%)		41 (43.62%)	55 (62.50%)	34 (57.63%)	32 (68.09%)	30.42 (P = 0.001^*^)	1.00		1.00	1.00	–		–	–
GA		17 (38.64%)	36 (41.86%)		38 (40.43%)	29 (32.95%)	24 (40.68%)	14 (29.79%)		1.47 [0.90–2.96]		1.32 [0.74–2.36]	6.14 [1.94–19.71]	0.21		0.35	0.002^*^
AA		5 (11.36%)	11 (12.79%)		15 (15.96%)	4 (4.55%)	1 (1.69%)	2 (4.26%)		2.73 [0.89–8.37]		7.73 [1.65–36.32]	2.06 [1.12–3.78]	0.08		0.01^*^	0.02^*^

V. Distribution according to severity of airflow obstruction in COPD																	
Genotype		Mild (GOLD 1) (n = 61)	Moderate (GOLD 2) (n = 67)	Severe (GOLD 3) (n = 52)	Very severe (GOLD 4) (n = 44)	Control				A	B	C	D	A	B	C	D
GG	–	30 (49.18%)	33 (49.25%)	21 (40.39%)	18 (40.90%)	121 (62.37%)			28.00 (P = 0.001^*^)	1.00	1.00	1.00	1.00	–	–	–	–
GA		26 (42.63%)	28 (41.79%)	20 (38.46%)	17 (38.64%)	67 (34.54%)				1.55 [0.87–2.78]	1.52 [0.85–2.72]	1.73 [0.94–3.19]	1.69 [0.92–3.08]	0.14	0.16	0.07	0.09
AA		5 (8.20%)	6 (8.95%)	11 (21.15%)	9 (20.45%)	6 (3.09%)				3.37 [0.85–13.40]	3.80 [0.97–14.78]	10.85 [3.04–38.77]	10.08 [2.81–36.12]	0.08	0.05	0.0002^*^	0.0004^*^

Significant associations are shown in asterisk*.

CI, confidence interval; MAF, Minor allele frequency in control group; OR, Odds ratio.

A- OR against GOLD 1 patient vs control and its corresponding P value.

B- OR against GOLD 2 patient vs control and its corresponding P value.

C- OR against GOLD 3 patient vs control and its corresponding P value.

D- OR against GOLD 4 patient vs control and its corresponding P value.

Generalized Linear Model (GLM) analysis indicated that old age, disease severity, and rural residence were positively associated with the rs3741240 AA risk genotype. The detailed findings of the GLM analysis are presented in **Table 4**.

**Table 4:** Generalized linear model (GLM) analysis to assess the impact of different demographic parameters on risk genotypes of *SCGB1A1* rs3741240 (^*^P value significant).

Demographic parameter	Estimate	Std. error	t-value	P-value
Disease Severity	0.71	0.10	6.90	<0.00001^*^
Age	0.04	0.01	3.71	0.0004^*^
Demographic parameter	Estimate	Std. Error	z-value	P-value
Residence	1.59	0.46	3.46	0.001^*^

### FEV_1_ and SNP genotypes

**Figure 2:**
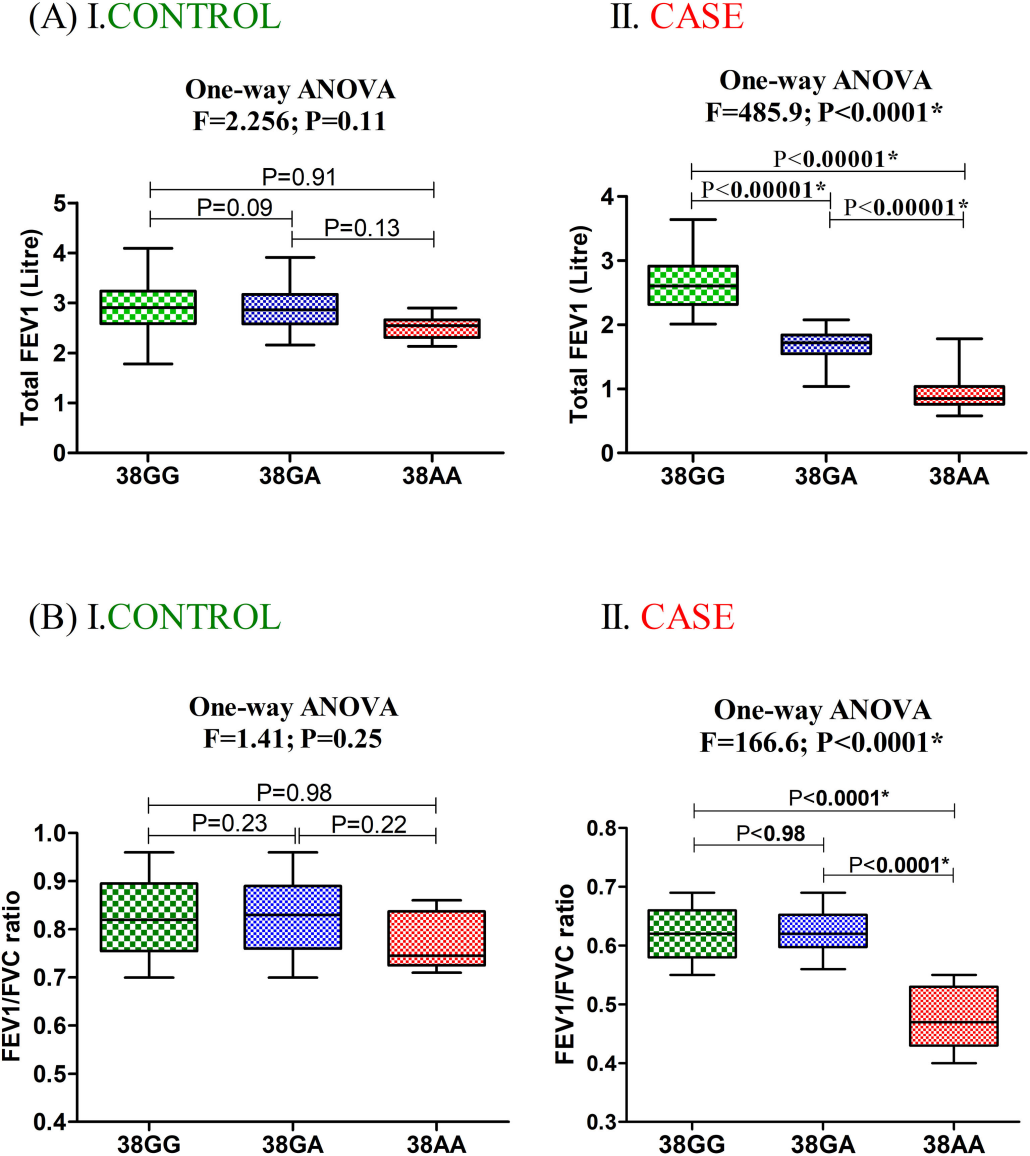
Level of **(A)** FEV1 and **(B)** FEV1/FVC ratio in case and control population bearing different genotypes for *SCGB1A1* rs3741240. (* P value significant).

The association of FEV_1_ and FEV_1_/FVC ratio with cases and controls bearing the rs3741240 polymorphism is shown in **Figure 2**. One-way ANOVA revealed that a significant difference existed in FEV_1_ and FEV_1_/FVC ratio of patients bearing polymorphic genotypes (F = 485.9, P <0.0001; F = 166.6, P <0.0001, respectively), while those factors were non-significant in the control population (F = 2.26, P = 0.11; F = 1.41, P = 0.25, respectively). Both FEV1 (P <0.00001) and FEV1/FVC (P <0.0001) were considerably lower in patients with AA risk genotypes.

### Localization of CC16 protein in bronchial epithelial cells

To localize the CC16 protein in BECs, Giemsa and immunofluorescence staining techniques were used. Giemsa staining and brightfield images of BECs provided an initial morphological assessment of the bronchial epithelial cell monolayer (**Figures 1C, D**). As shown in **Figure 1C**, cytoplasmic granularity was clearly observed in a subset of cells. Immunofluorescence staining revealed granular and cytoplasmic staining with no significant nuclear signal. The nuclei were counterstained with DAPI to allow a clear distinction between the nuclear and cytoplasmic compartments (**Figure 1E**).

### SCGB1A1 mRNA expression in polymorphic variants via qRTPCR analysis

Quantitative real-time PCR analysis revealed that SCGB1A1 mRNA expression was significantly decreased in BECs from COPD patients compared to healthy controls (**Figure 1F**). One-way ANOVA revealed significant differences in expression between patients with polymorphic genotypes and controls (F = 304.5; P <0.0001). The *Post Hoc* Tukey test with Bonferroni correction showed significant differences in *SCGB1A1* expression between 38GA (P = 0.001) and 38AA mutant genotypes (P = 0.0001) and the wild-type 38GG genotype in COPD patients. The lowest expression level was observed in patients with 38AA SNPs. No significant difference in *SCGB1A1* expression was observed between patients with the wild 38GG genotype and healthy controls (p = 0.59), suggesting that SCGB1A1 downregulation is associated with different rs3741240 genotypes in COPD patients.

### Assessment of CC16 protein expression by Western blot

Western blot analysis of CC16 protein levels showed a significant reduction in COPD patients with different polymorphic genotypes compared to healthy controls. The intensity of the CC16 band was markedly reduced in COPD patients compared to that in controls (**Figure 1G**).

Statistical analysis using one-way ANOVA showed a significant difference in CC16 protein expression among the study groups (F = 1937, *P <*0.0001). *Post hoc* comparisons using Tukey’s test with Bonferroni correction demonstrated that patients with the 38AA risk genotype exhibited significantly lower CC16 expression levels than those with the 38GA and 38GG genotypes (*P <*0.00001) and healthy controls (*P <*0.00001) (**Figure 1H**).

### *In silico* study of *SCGB1A1* polymorphic variants

#### Analyses on alteration of miRNA–target interactions due to SNP in *SCGB1A1* promoter

Our study investigated the impact of an SNP in the *SCGB1A1* promoter on miRNA-mediated regulation of *SCGB1A1* expression. In the current study, we identified 13 miRNAs targeting the 5′ UTR of *SCGB1A1* from miRDB (**Table 5**).

**Table 5:** Details of 13 predicted miRNAs from miRDB.

miRNA name	Target score		Seed location	
	Wild type	Mutant	Wild type	Mutant
hsa-miR-340-3p	82	76	112, 124	112
hsa-miR-6827-3p	80	67	112, 125	112
hsa-miR-654-5p	80	80	98	98
hsa-miR-541-3p	80	80	98	98
hsa-miR-6804-5p	72	72	169	169
hsa-miR-4653-5p	71	72	108, 217	108, 217
hsa-miR-3921	71	72	108, 217	108, 217
hsa-miR-765	63	63	145	145
hsa-miR-5088-5p	62	62	16	16
hsa-miR-2277-3p	61	61	163	163
hsa-miR-500b-3p	59	59	41	41
hsa-miR-1301-5p	54	54	55	55
hsa-miR-11181-3p	50	51	145	145

**Figure 3:**
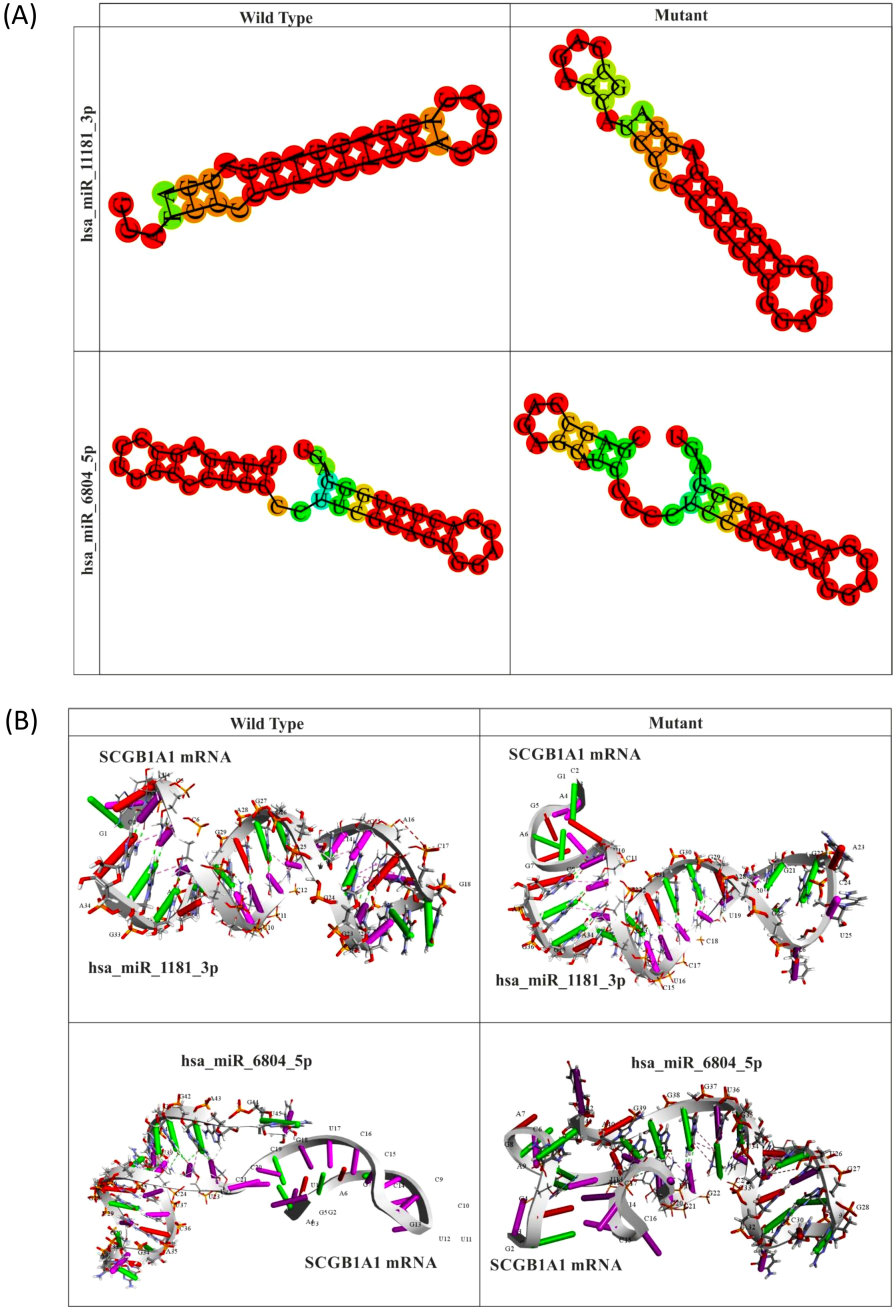
**(A)** The optimal secondary structures of both wild type and mutant miRNA-mRNA (*SCGB1A1*) complexes predicted by RNAfold web server. **(B)** Predicted 3D structures of both wild-type and mutant mRNA–miRNA complexes for the two microRNA-target interactions (MTIs), generated using the RNA COMPOSER server.

These 13 miRNAs were analyzed using RNA22 v2 to otain MTI details (**Table 6**). Among these 13 miRNAs, two miRNAs (hsa_miR_11181_3p and hsa_miR_6804_5p) bound in the vicinity of the mutation site. The binding affinity of these two miRNAs with both wild-type and mutant *SCGB1A1* promoter was measured in terms of their corresponding folding energy in kcal/mol from RNA 22v2. As shown in **Table 6**, hsa-miR-11181-3p exhibited a stronger binding affinity for the mutant promoter than for the wild-type (WT) promoter, whereas hsa-miR-6804 demonstrated a higher binding affinity for the wild-type *SCGB1A1* promoter than for its mutant counterpart. The predicted dot bracket notations (**Table 6**) and optimal secondary structures (**Figure 3A**) of the two miRNA–mRNA duplexes, each corresponding to the WT and mutant promoters, were obtained using the RNAfold web server.

**Table 6:** The MTI details of two miRNAs from RNA22 v2.

miRNAs targeting SCGB1A1 5’ UTR	RNA 22v2				RNAfold	
	Wild type		Mutant		Wild type	Mutant
	Folding energy (Kcal/mol)	p value	Folding energy (Kcal/mol)	p value	Dot bracket notation	Dot bracket notation
hsa_miR_11181_3p	−20	0.0645	−21.3	0.0645	…(((.(((((((((….))))))))).)))	((….)).(((.(((((((……))))))).)))
hsa_miR_6804_5p	−13.8	0.0244	−12	0.0645	.((((.((….)).)))).((((((((….))))))))…	.((((….)).))….((((((((….))))))))…

The 3D structures of the WT and mutant miRNA–mRNA (*SCGB1A1*) complexes were predicted using the RNA COMPOSER server, with the corresponding dot-bracket notations of the miRNA–mRNA duplexes provided as inputs (**Figure 3B**).

#### Analyses of molecular docking between mRNA–miRNA duplexes and hAGO2 protein

**Figure 4:**
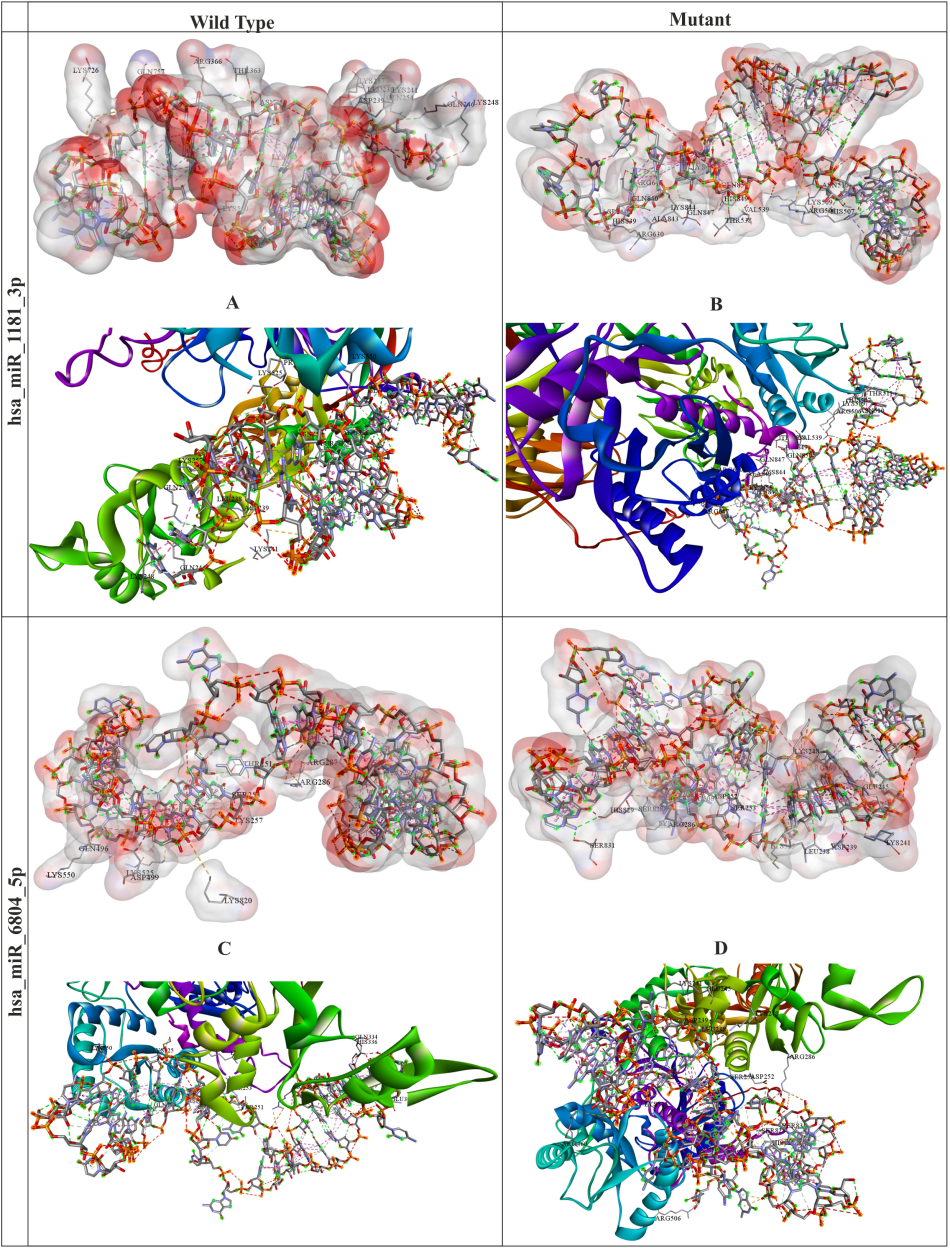
Molecular interactions of wild-type and mutant miRNA–mRNA–hAGO2 complexes. Panels **(A, B)** depict hsa_miR_1181_3p, while panels **(C, D)** show hsa_miR_6804_5p.

To investigate the mechanism underlying the alteration in gene expression due to SNP-induced changes in the binding affinity of the targeted miRNAs to *the SCGB1A1* promoter, we performed molecular docking of pre-prepared, cleaned. Energy-minimized human Argonaute 2 (hAGO2) with these two mRNA–miRNA duplexes, each in WT and mutant forms, was used with the HDOCK web server (**Table 7**, **Figure 4**). Consistent with the earlier observation of a higher binding affinity of hsa-miR-11181-3p to the mutant promoter, the results presented in **Table 7** also support this finding, showing that the miRNA (hsa-miR-11181-3p)–target (mutant *SCGB1A1* promoter) duplex exhibited a stronger binding affinity with hAGO2 than its WT counterpart. A similar trend was observed for hsa-miR-6804-5p, consistent with previous results.

**Table 7:** Docking scores along with confidence scores of WT miRNA-mRNA-AGO2 and mutant miRNA–mRNA–AGO2 complexes for both MTIs.

Model	Wild type		Mutant	
	Docking score	Confidence score	Docking score	Confidence score
hsa_miR_11181_3p VS AGO2	−308.38	0.9596	−340.11	0.9782
hsa-miR-6804-5p VS AGO2	−304.44	0.9564	−300.01	0.9526

#### Analyses on alteration of transcription factors-target (promoter) interactions due to SNP in *SCGB1A1* promoter

**Figure 5:**
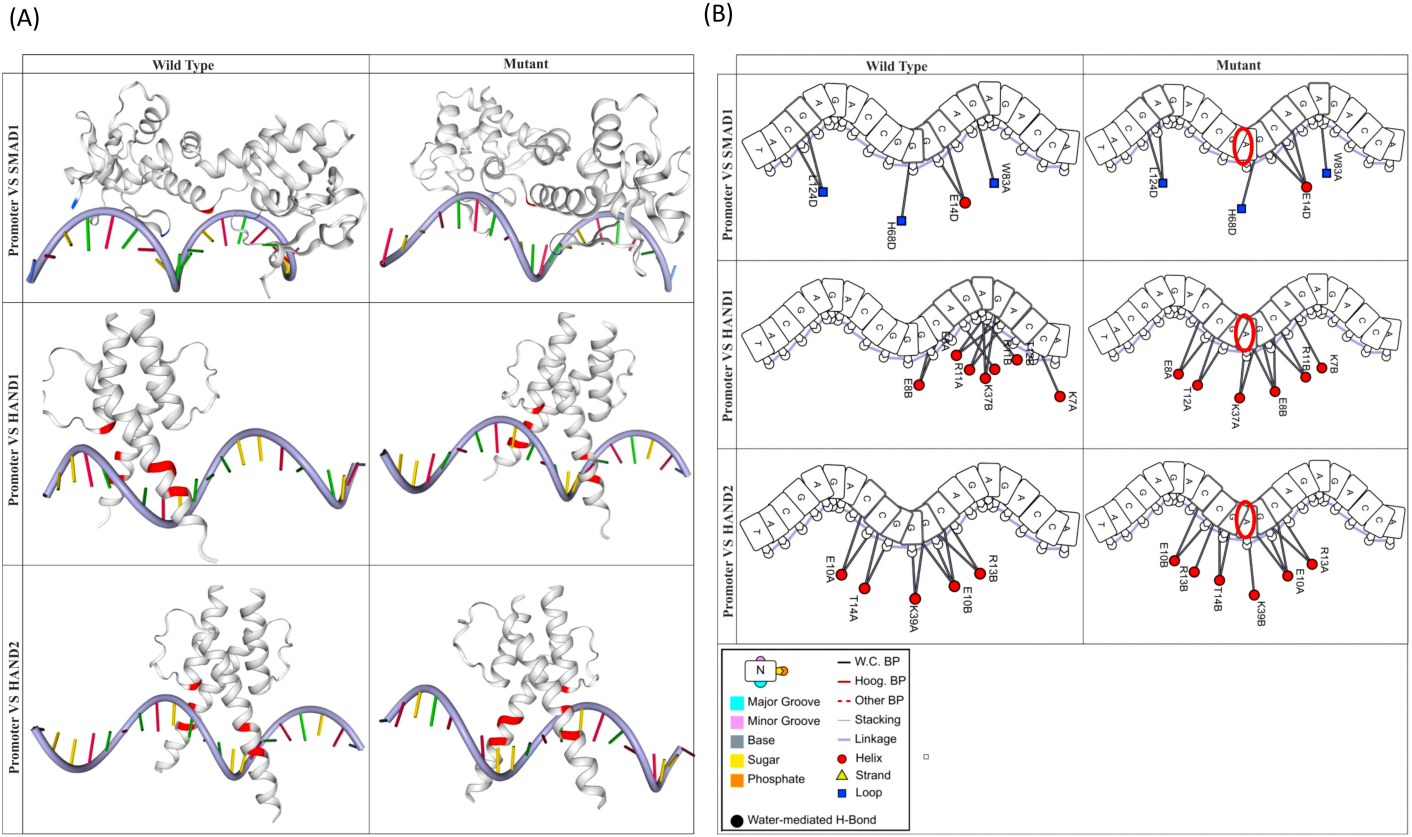
**(A)** Illustration of docking poses of 3 TF-promoter complexes. **(B)** Interacting interfaces of 3 TF-promoter complexes.

In this study, we have identified three TFs (SMAD1, HAND1, and HAND2) that target the ACCAGAGACGGGCCAGAGCAT sequence of *the SCGB1A1* promoter using the hTFtarget web server. The details of the DNA-binding domains of these three TFs, obtained from UniProt are presented in **Table 8**. The DNA-binding domains of these three TFs were modeled using template-guided homology modeling. GalaxyGemini dimerized the modeled structures, as dimerization is known to enhance the DNA-binding affinity and specificity of transcription factors (27). The selected nucleotide stretch (ACCAGAGACGGGCCAGAGCAT) of the promoter (both WT and mutant) was constructed using Discovery Studio. To investigate whether an SNP in the promoter region can alter the binding affinity of a transcription factor (TF), we performed molecular docking analyses using the HDOCK web server. The DNA-binding domains of the three selected TFs were docked to the modeled *SCGB1A1* promoter in both the wild-type and mutant forms (**Table 9**, **Figure 5A**). **Table 9** shows that the mutant promoter of *the SCGB1A1* gene did not alter the binding affinity of these three TFs. The protein (TFs)—DNAproDB analyzed DNA (*SCGB1A1* Promoter) interactions for both WT and mutant promoters. The docking poses and interacting interfaces of the TF-promoter complexes are shown in **Figures 5A, B**.

**Table 8:** Details of DNA binding domains of three TFs.

Information types	SMAD1	HAND1	HAND2
DNA binding Domain	MH1	bHLH	bHLH
Residue position (amino acid) of domain	12-136	96-146	99-151
UniProt ID	Q15797	O96004	P61296

**Table 9:** Docking and confidence scores for the interactions between wild-type and mutant *SCGB1A1* promoters and the three transcription factors.

Model	Wild type		Mutant	
	Docking score	Confidence score	Docking score	Confidence score
Promoter VS SMAD1	−260.19	0.9006	−257.23	0.8952
Promoter VS HAND1	−303.81	0.9559	−299.94	0.9525
Promoter VS HAND2	−325.42	0.9709	−316.56	0.9655

## Discussion

Chronic Obstructive Pulmonary Disease (COPD) is a complex, multifactorial respiratory condition characterized by persistent airflow limitation, chronic inflammation, and progressive lung tissue damage (28, 29). In 2015, COPD accounted for approximately 3.2 million deaths worldwide, making it the third leading cause of death among older adults (28). Its chronic nature and high prevalence place a substantial burden on healthcare systems, particularly in the aging population (29). Our study confirmed that COPD was significantly more common in individuals aged >50 than in those aged 20–50 years (**Table 3**). A global study by Wang et al. (30) reported that COPD prevalence increases steadily with age, peaking in individuals aged 70–74 years, with the highest mortality rates among those aged 80 and older. Similarly, Weeks et al. (31) found that COPD prevalence rose from 0.4% in those aged 18–24 years to 10.5% in individuals aged 75 years and older.

While environmental factors such as smoking remain the principal risk factor, growing evidence supports the contribution of genetic variants in modulating individual susceptibility to COPD (5). Among these candidates, the *SCGB1A1* gene polymorphism rs3741240 has emerged as a significant contributor to disease progression, primarily through its downregulatory effect on CC16 expression, a critical anti-inflammatory and immunomodulatory protein secreted by club cells in the airway epithelium (6).

The *SCGB1A1* gene, located on chromosome 11q12.3, encodes CC16, also known as Clara Cell Secretory Protein or uteroglobin (7). CC16 plays a protective role in the lungs by modulating inflammatory responses, neutralizing oxidative stress, and maintaining epithelial integrity (32). Additionally, epigenetic modifications, such as promoter hypermethylation of the *SCGB1A1* gene in COPD patients, contribute to reduced gene expression and compromise its anti-inflammatory function (8). Reduced CC16 levels have been consistently associated with impaired pulmonary function, increased epithelial permeability, and heightened inflammatory responses in COPD patients (33). In the present study, 64.73% of the participants reported a family history of COPD (maternal, paternal, or both), further supporting a genetic predisposition to the disease (**Table 2**). Notably, a Mendelian randomization study using *SCGB1A1*-linked SNPs demonstrated that genetically elevated CC16 levels causally reduce the risk of developing COPD and slow its progression (32). We observed, for the first time in an Indian population, a significant association between the *SCGB1A1* rs3741240 polymorphism and COPD risk. Among the genetic variants within the *SCGB1A1* locus, rs3741240, located in the 5′ untranslated region (5′ UTR), has been the most extensively studied (13). The rs3741240 SNP (G > A, also known as G38A) has been reported to correlate with lower CC16 expression levels at both the mRNA and protein levels, supporting a transcriptional and post-transcriptional impact (34). The present study revealed that COPD patients carrying the rs3741240 AA risk genotype had a significantly higher risk of developing COPD (**Table 3**). Interestingly, female patients with the risk genotype were at greater risk than male patients. This aligns with the findings of Han et al. (35), who reported that, for the first time in 2000, the number of women dying from COPD in the United States surpassed the number of male deaths. Furthermore, Tam et al. (36) suggested that female sex hormones influence airway inflammation, mucus production, and other biological processes associated with COPD in women.

The higher COPD risk observed among older individuals with the AA genotype suggests that age-related physiological decline may compound the genetic susceptibility conferred by the SCGB1A1 variant. This observation is consistent with that of a study by Stone et al. (37), who identified increasing age as a significant risk factor for COPD management. Moreover, the increased prevalence of COPD among individuals with the AA genotype residing in rural and semi-urban areas highlights the potential influence of environmental exposures. This finding supports the previous research by Raju et al. (38), who reported that urban-rural disparities in COPD prevalence may be attributed not only to smoking and indoor air pollution but also to occupational and agricultural exposure. Consistent with this, an Indian hospital-based study by Agarwal et al. (39) reported that 75.4% of rural female biomass fuel users had COPD compared to non-users. Together, these observations emphasize the interplay between genetic predisposition and environmental risk factors in shaping COPD susceptibility in the Indian population. Furthermore, the greater frequency of the AA genotype among patients in advanced GOLD stages (3 and 4) indicates that this variant may contribute not only to COPD susceptibility but also to disease progression and severity.

The association of the 38AA genotype with reduced lung function parameters underscores its potential role in compromising airway integrity, possibly through reduced CC16-mediated epithelial protection. In the control group, individuals with all three genotypes generally exhibited only mild-to-moderate COPD associated with the rs3741240 variant. In contrast, among COPD patients, those harboring the 38AA risk genotype predominantly developed severe disease. In contrast, individuals with heterozygous or major genotypes were more likely to exhibit mild-to-moderate COPD severity (**Figure 2**).

The localization of CC16 in bronchial epithelial cells (BECs) highlights its potential role in airway homeostasis and epithelial cell function (40). Giemsa staining revealed cytoplasmic granularity in a subset of cells, suggesting active secretion and a secretory phenotype (41). This observation was further supported by immunofluorescence analysis, which confirmed the cytoplasmic localization of CC16 in BECs with a distinct granular pattern, consistent with its known function as a secretory protein (42). The absence of nuclear staining emphasizes the cytoplasmic confinement of CC16 activity (42). Moreover, the downregulation of CC16 removes a critical inhibitory checkpoint in the immune response, facilitating chronic neutrophilic inflammation, oxidative injury, and structural damage to the alveolar walls (41). These findings align with previous reports identifying Clara cells as the primary source of CC16 and support its role as a biomarker of epithelial integrity and inflammation in airway diseases such as COPD (43).

We demonstrated that SCGB1A1 expression was downregulated in individuals carrying the 38GA or 38AA genotypes at rs3741240 compared to the control group with the 38GG genotype, as assessed by RT-PCR and western blot analyses. The presence of the rs3741240 risk allele may serve as a molecular biomarker to identify individuals with heightened susceptibility to COPD, even in non-smokers, suggesting a genotype-driven endotype of this disease. These findings are consistent with those of Li et al. (34), who reported that the A risk allele of rs3741240 was significantly associated with lower SCGB1A1 mRNA expression in both sputum and lung tissues, with regression slopes of β = −0.81 (P = 0.007) in cross-sectional and β = −0.69 (P = 0.04) in longitudinal cohorts. Moreover, another study showed a clear allelic imbalance, with excess expression of the G allele over A (P <10^−6^), suggesting that the A allele leads to reduced transcriptional activity (44). These findings align with protein-level observations: individuals with A allele genotypes (AG or AA) show lower CC16 levels in various biological fluids, as first reported by Kim et al. and others (13, 45). Kim et al. (13) observed that the minor allele at this SNP was negatively associated with SCGB1A1 mRNA expression levels in sputum samples. In an adult cystic fibrosis cohort, rs3741240 explained up to 19% of the variance in serum CC16; each A allele was associated with a roughly −0.63 SD decrease (P ≈ 6.2 × 10^−18^) (46). This aligns with a broader neonatal/adult population study, in which rs3741240-A correlated with reduced CC16 from birth through early mid-adulthood (47). Therefore, it can be said that the rs3741240 polymorphism is associated with allele-specific downregulation of CC16 at both the transcriptional and translational levels in bronchial brush cells from the population of West Bengal, India. Notably, this also opens avenues for targeted therapeutic strategies, such as gene therapy to restore CC16 expression or recombinant CC16 protein supplementation, to mitigate inflammation and epithelial injury.

In addition to wet-lab experimentation, bioinformatics analyses were employed to reinforce the hypothesis that the rs3741240 SNP may regulate CC16 expression and play a role in COPD pathogenesis. This approach was based on the premise that numerous bioinformatics studies have successfully established associations between specific SNPs and the development of various diseases (48–50). SNPs can modulate gene expression by altering the binding affinity of TFs or miRNAs to target gene sequences (51–53). Therefore, SNP rs3741240 in the *SCGB1A1* gene (which encodes CC16) may alter its expression by altering TF or miRNA binding, thereby contributing to disease development. Previous studies have demonstrated that SNPs can influence transcription factor (TF) binding, thereby altering gene expression (54, 55). In the present study, three transcription factors, SMAD1, HAND1, and HAND2, exhibited a slightly better binding affinity for the polymorphic *SCGB1A1* promoter than their wild type counterparts. Since this observation alone does not elucidate the mechanism underlying TF-mediated modulation of CC16 expression, we further extended our bioinformatics investigation to explore miRNA-mediated regulation of CC16 expression affected by the rs3741240 SNP. Accordingly, two miRNAs (hsa_miR_11181_3p and hsa_miR_6804_5p) were predicted to bind in proximity to the rs3741240 locus. Among them, hsa-miR-11181-3p appears to downregulate CC16 expression through AGO2-mediated silencing of a polymorphic variant of *SCGB1A1*. The role of miRNAs in AGO2-mediated gene silencing has been reported in several studies (56, 57). It should be noted that the present study is limited to computational analyses. Therefore, further wet-lab experiments are necessary to confirm TF- and miRNA-mediated silencing of CC16.

Reduced CC16 expression suppresses the epithelial-derived antimicrobial protein Short Palate, Lung, and Nasal Epithelial Clone 1 (SPLUNC1) through impairment of downstream signal transduction mediated by the CC16 receptor, Late Antigen-2 (VLA-2), on epithelial cells. Additionally, diminished CC16-VLA-4 signaling in leukocytes results in decreased leukocyte adhesion to endothelial cells, thereby promoting lung and airway inflammation (58, 59). Similarly, in the present study, the reduced CC16 expression associated with the rs3741240 SNP may contribute to COPD pathogenesis by compromising the respiratory host defense system.

However, the present study had a few limitations. The sample size for specific genotypic subgroups was limited, which may have affected the statistical robustness. Moreover, the study design was correlative and did not include functional assays to confirm the mechanistic role of the rs3741240 variant directly. Although previous Mendelian randomization studies using SCGB1A1-linked SNPs have demonstrated that genetically elevated CC16 levels may causally reduce COPD risk and progression, our study provides the first evidence of a significant association between the SCGB1A1 rs3741240 polymorphism and COPD susceptibility in the Indian population. This causal relationship requires verification through functional validation in larger, multicenter cohorts.

In conclusion, the *SCGB1A1* rs3741240 variant downregulates CC16 expression, contributing to COPD pathogenesis, possibly by promoting unchecked inflammation and epithelial damage. The elucidation of this molecular axis provides valuable insights into disease mechanisms and highlights potential clinical applications. Targeting this pathway, either by restoring CC16 levels or modulating *SCGB1A1* activity, may offer promising avenues for developing specific personalized medicine-based therapeutic approaches and novel treatment strategies for COPD management.

## Data Availability

The data presented in the study are deposited in the ClinVar repository, accession number SCV007106175.
